# Utility and Evaluation of Applied Project Management Processes Within a Large Multicountry Health Systems Development Project Conducted During the Coronavirus Disease 2019 (COVID-19) Pandemic

**DOI:** 10.1093/cid/ciad549

**Published:** 2023-12-20

**Authors:** Soo Young Kwon, Sanjay Gautam, Kritika Poudel, Hasini Banneheke, Delfim Ferreira, Manish Gautam, Michelle Hau'ofa, Nashmia Mahmood, Bouahome Phommalad, Mohammad Julhas Sujan, Pema Yangzom, Hea Sun Joh, Alina Shaw, Brooke Dolabella, Hye Jin Seo, Jong-Hoon Kim, Partick Gallagher, William R MacWright, Nimesh Poudyal, Florian Marks, Marianne Holm

**Affiliations:** International Vaccine Institute, Seoul, Republic of Korea; International Vaccine Institute, Seoul, Republic of Korea; Research and Collaboration, Anka Analytica, Melbourne, Australia; Research and Collaboration, Anka Analytica, Melbourne, Australia; Faculty of Medical Sciences, University of Sri Jayewardenepura, Boralesgamuwa, Sri Lanka; National Directorate of Pharmacy and Medicines, Ministry of Health, Díli, Timor-Leste; Public Health Research, Anweshan Private Limited, Lalitpur, Nepal; The CAPTURA Project, Port Moresby, Papua New Guinea; UNDP, University of Illinois, Illinois, USA; Food and Drug Department, Ministry of Health, Vientiane, Lao People's Democratic Republic; International Vaccine Institute, Seoul, Republic of Korea; National Medical Service, Royal Government of Bhutan, Thimpu, Bhutan; International Vaccine Institute, Seoul, Republic of Korea; Public Health Surveillance Group, LLC, Princeton, New Jersey, USA; Public Health Surveillance Group, LLC, Princeton, New Jersey, USA; International Vaccine Institute, Seoul, Republic of Korea; International Vaccine Institute, Seoul, Republic of Korea; Public Health Surveillance Group, LLC, Princeton, New Jersey, USA; Public Health Surveillance Group, LLC, Princeton, New Jersey, USA; International Vaccine Institute, Seoul, Republic of Korea; International Vaccine Institute, Seoul, Republic of Korea; Cambridge Institute of Therapeutic Immunology and Infectious Disease, University of Cambridge School of Clinical Medicine, Cambridge, UK; Heidelberg Institute of Global Health, University of Heidelberg, Germany; Madagascar Institute for Vaccine Research, University of Antananarivo, Madagascar; International Vaccine Institute, Seoul, Republic of Korea

**Keywords:** CAPTURA, COVID-19 pandemic, project management, impact, antimicrobial resistance

## Abstract

The increasing trends in antimicrobial resistance (AMR) continue to pose a significant threat to human health, with grave consequences in low- and middle-income countries. In collaboration with local governments and microbiology laboratories in South Asian and Southeast Asian countries, the Capturing Data on Antimicrobial Resistance Patterns and Trends in Use in Regions of Asia (CAPTURA) project worked to identify gaps and expand the volume of existing AMR data to inform decision-makers on how to best strengthen their national AMR surveillance capacity. This article describes overall project management processes and the strategies implemented to address the disruptive impact of the coronavirus disease 2019 (COVID-19) pandemic on the project activities across diverse contexts in different countries. Also, it assesses in-country team's feedback on the conduct of activities and their overall impact on project completion. The strategies employed were tailored to the specific context of each country and included increased communication and collaboration among consortium partners and in-country teams, as well as hiring of additional in-country team members. This paper highlights the importance of local representation and capacities as well as real-time (virtual) engagement with stakeholders, ensuring close monitoring of the local situation and ability to tailor context-specific mitigation strategies to continue project implementation during disruptive external circumstances.

## Teaser

We describe the overall project management processes, as well as the strategies implemented to address the disruptive impact of the COVID-19 pandemic on the CAPTURA project activities across diverse contexts in different countries.

Antimicrobial resistance (AMR) has emerged as a dominant global public health threat with increased morbidity, mortality, and healthcare expenditures worldwide, requiring urgent multisectoral action [1]. Although identified as an important part of the strategy to combat AMR, surveillance of AMR patterns and trends has remained relatively limited and fragmented in most of the low- and middle-income countries (LMICs) across Africa and Asia [2]. Furthermore, the ongoing coronavirus disease 2019 (COVID-19) pandemic posed an unprecedented challenge to implementing health programs and severely impacted AMR surveillance activities due to movement restrictions, diverted personnel and resources to minimize the spread of the pandemic, and overwhelmed healthcare systems. A survey from 73 Global Antimicrobial Resistance Surveillance System (GLASS) countries found a significant impact of COVID-19 on AMR surveillance, prevention, and control and highlighted the serious need to continuously ensure AMR as a global health priority [3].

The “Capturing Data on Antimicrobial Resistance Patterns and Trends in Use in Regions of Asia” (CAPTURA) is a multicountry development project tasked with identifying and collating retrospective data on AMR, antimicrobial consumption (AMC), and antimicrobial use (AMU) in several LMICs across South and Southeast Asia. In collaboration with local governments and microbiology laboratories, CAPTURA worked to identify gaps and expand the AMR/AMC/AMU data collection to inform decision-makers on how to best strengthen their national surveillance capacity [4].

In this article, we evaluate the continuously updated risk assessment records and associated mitigation approaches implemented during COVID-19, as well as present the overall project management processes applied to the CAPTURA program during the implementation phase. We discuss and evaluate how these measures were utilized to mitigate the impact of the pandemic on activities across diverse contexts in different countries, incorporating assessments and feedback provided by in-country teams. Additionally, we examine the overall effect of these mitigations on project delay and completion.

## CAPTURA PROJECT MANAGEMENT PROCESSES

The CAPTURA project worked to identify gaps and expand AMR/AMC/AMU data collection. This included creating a Country Implementation Plan for each country, which was informed by metadata collection through a desktop review, key informant interviews, and questionnaires and engaging with both AMR Country Coordinators and data owners. AMR, AMU, and AMC data were collected, curated, and analyzed during the project.

To ensure effective project implementation, the lead grantee put in place project management principles to cover various aspects of the project life cycle, such as initiation of the project, monitoring and reporting progress on an monthly basis, managing risks, addressing change request, and closing. These principles were designed to provide a structured and proactive approach to managing the project, enabling the project team to stay on key issues and make proper decisions under institutional governance throughout the project's life cycle.

During the COVID-19 pandemic period, a separate COVID-19 report was generated to identify and set different mitigation strategies and record them in the table on a weekly basis (initially), followed by a bi-monthly and monthly basis. In addition, the CAPTURA team based at the International Vaccine Institute (IVI) in Korea increased communication with representatives from member countries for risk assessment. The regularly updated record was analyzed at the end of the project for evaluating situation and mitigation approaches implemented during the COVID-19 pandemic. The report was also regularly shared with the funder's managing agency for their awareness and tracking of delays/progress. Over time, the project hired and relied on more in-country team members and prioritized communication among partners and in-country teams. Another aspect of the mitigation plan was identifying sustainable activities and utilizing online monitoring and trainings. The project timeline, activities, and budget were periodically reviewed and modified in response to challenges and circumstances.

## CHALLENGES AND MITIGATION APPROACHES DURING THE COVID-19 PANDEMIC

With the varying situation and national strategies to respond to COVID-19 pandemic, CAPTURA could not keep the same approach for all countries. Therefore, different mitigation strategies were implemented to continue CAPTURA activities based on communication with in-country team and consortium partners. While border closure, lockdown, curfews, social distancing, and strict quarantine measures were in place, the CAPTURA project was able to continue to identify sustainable activities and execute data-related activities without interfering with the local outbreak response and public safety.

Based on the CAPTURA team activities, the mitigation plans were divided into 2 phases.

During phase 1 (1 December 2019 to 30 April 2020), the team recognized COVID-19 to be a major threat to the proper conduct of CAPTURA and developed a register of related risks. During phase 2 (1 May 2020 to 31 May 2022), the team implemented the mitigation plans that were formulated in phase 1 to address the risks posed by the pandemic. Furthermore, during the development of this paper, short questionnaires (3 questions) were developed to receive subjective recount on CAPTURA project implementation challenges during COVID-19 pandemic and suggestions for future reference. The questionnaires were sent to in-country team members via email. Two weeks’ time was given to complete the questionnaire, and members were followed up 3–4 times via email. Among representatives from 9 countries, 8 responded to the questionnaire.

**Figure 1.**
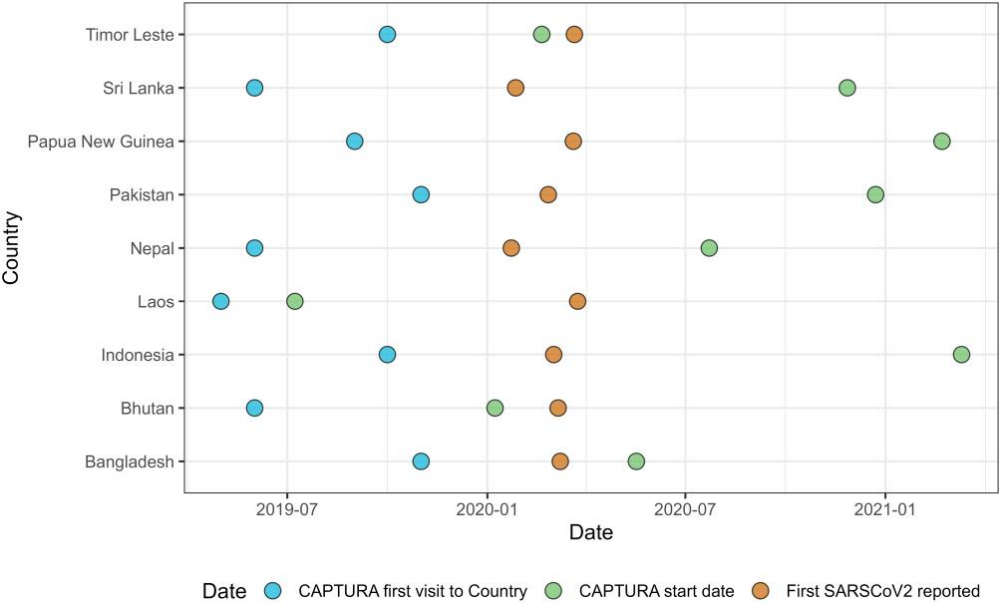
Phases of Capturing Data on Antimicrobial Resistance Patterns and Trends in Use in Regions of Asia (CAPTURA) project in different countries. Phases of CAPTURA project: Starting point is the date of the first data transfer agreement (DTA) signed or government approval obtained. Date of approval by government. For Sri Lanka and Pakistan, the date represents the day the first DTA was signed. For Indonesia, the agreement between CAPTURA/Ministry of Health to carry over antimicrobial resistance/antimicrobial use questionnaires is the date. Scopes for these countries are limited. Abbreviations: CAPTURA, Capturing Data on Antimicrobial Resistance Patterns and Trends in Use in Regions of Asia; SARS-CoV-2, severe acute respiratory syndrome coronavirus 2.

The COVID-19 pandemic challenged and pushed the process of data surveys from in-person to remote, mobilizing researchers and practitioners alike to learn new systems, adapt to the mitigating strategies, and engage in a great deal of trial and error. Figure 1 presents the dates and different work phases of the CAPTURA team during the COVID-19 pandemic. Due to government officials being occupied with the COVID-19 response, the project experienced a significant decline in communication with major stakeholders in the countries. Also, all planned on-site assessments and trainings were mostly canceled and/or delayed. For example, in March 2020, all scheduled trainings and assessments in Indonesia, Timor-Leste, and Pakistan were canceled, and instead, they were conducted virtually over the next 1–3 months. To address this challenge, CAPTURA proactively hired a local team to liaise with in-country stakeholders and started to reform all meetings and trainings to virtual. In addition, online tools and applications were adopted and centralized data repository was set to minimize the disruptions in communication channels and workflow.

CAPTURA's initial data collection plan was interrupted by COVID-19 measurements across all countries. The CAPTURA in-country team members were encouraged to strictly follow guidelines to support the containment of COVID-19 pandemic in their settings. Therefore, during phase 2, according to the circumstantial situations in individual countries, implementation strategies were tailed to continue CAPTURA activities. Since the consortium consisted of partners based across the world, the use of virtual platforms for communication was already well established. However, the in-country team had to find innovative ways to engage and fulfill their planned activities virtually. This included identifying additional local consultants, and planning for virtual and/or amended laboratory assessments. Meanwhile, identifying data sources for AMR/AMU/AMC surveillance and capacity-building activities, such as providing training for WHONET database software and Epicollect use, continued as far as possible. However, overall progress was delayed at least 3–6 months and was primarily related to challenges with virtual engagement of stakeholders such as data owners. The switch from in-person interaction to web-based platforms was impacted by different time zones and limited availability of reliable internet and electricity supplies—delaying facility mapping and data collection activities.

Monitoring activities led by the Public Health Surveillance Group changed, too. Virtual monitoring was conducted; bi-weekly calls were made to track the progress in data collection. A framework of methods to guide virtual monitoring processes, compliance assessments, updated processes, and setting of corrective actions was prepared. This guided one point-in-time assessment per country and included areas such as data collection and compliance, capacity building, data sharing and dissemination, and detailed plans for future activities. As demonstrated, the CAPTURA project began implementing a country-tailored approach even before the COVID-19 pandemic and it was updated periodically based on the local COVID-19 situation and progress. For example, during the initial phase (January–April 2020), team members in Bangladesh started to identify data sources and in-country stakeholders, while Nepal's team initiated WHONET training and continued AMR data collection activities. Bhutan's laboratory assessment was slightly delayed due to its closed international border, strict country-imposed restrictions, and the limited availability of officials in the Ministry of Health (the CAPTURA grantee) during the pandemic. Overall, the close virtual monitoring of the local situation and tailored engagement with stakeholders led to the successful implementation of CAPTURA projects during unprecedented circumstances.

At IVI, CAPTURA countries were categorized into different tiers based on the extent of permissible activities, ranging from full activities to no activities. The budget revision was made to reflect the work scope per country and reallocate unspent travel budget to support increased costs in promoting online-based activities and hiring in-country teams. The project timeline was another area highly impacted by the COVID-19 pandemic. Engaging with in-country representatives was initially planned for 1 year, but delayed until 2 years after the start of program implementation. These changes were addressed, approved, and documented following IVI's institutional process and donor approval.

We gave each in-country representative short questionnaires (3 questions) to understand the real-time experience and feedback while working on the CAPTURA project during the pandemic. We received responses from 8 of the 9 countries contacted, outlining the challenges they faced and offering recommendations for the future. Table 1 presents the unaltered yet succinct versions of the received answers. Switching to a virtual platform for a wide range of activities (eg, meetings and trainings) and limited accessibility to facilities was a common denominator across different countries. While respondents from Bangladesh mentioned “not higher” impact of the COVID-19 pandemic in CAPTURA activities, there was low participation, difficulty attending hospitals, and long working hours in Nepal. Similarly, developing more robust technical facilities was a key recommendation from Bangladesh and the Lao People’s Democratic Republic, and effective stakeholder engagement was suggested as critical from Nepal, Sri Lanka, and Pakistan. A CAPTURA representative from Bhutan highlighted working on risk mitigation measures for similar projects in the future.

**Table 1.** Responses Received to Questionnaires by a Member of Participating Laboratories

**Question 1. How did COVID-19 impact the implementation of the CAPTURA project?**	
Bangladesh	“The impact on the CAPTURA activities was not higher in Bangladesh because of the close collaboration with the Ministry personnel and we were able to obtain a letter of travel authorisation and special permission to visit the laboratories. Our government partners gave us the utmost support while they assigned expected microbiologist and technical staff for the CAPTURA field activities in order to identify potential laboratories, pharmacies, conduct Rapid Laboratory Quality Assessment, build capacity, and data collection. Initially, we started conducting WHONET workshops in-person in different divisions and moved to a virtual working environment during the worst period of the COVID-19 pandemic. At the laboratory level, we virtually carried out remote installation, configuration, and refresher training for WHONET and began collecting data for antimicrobial susceptibility testing. Moreover, we developed a web-based live chatbot for each laboratory and shared the platform with the data entry person so that we were able to receive their urgent problems and fix them by tacking remote desktop access. Regarding the project's scoping and operations in Bangladesh, there was deficiency of time to interact with all the additional laboratories and pharmacies that subsequently expressed interest, as well as various government organizations and other international stakeholders because of several nation-wide lockdowns.”
Bhutan	“The implementation of the CAPTURA project and the budget were prepared during a pre-COVID situation. However, when the actual implementation began, the COVID outbreak affected the activities. Due to a series of lockdowns and outbreaks in the country, travel restrictions took place, and workshops and training were postponed. Further, the budget utilisation was reduced to 50% due to travel restrictions, and the per diem was decreased for the participants. In addition, due to travel restrictions, monitoring visits were cancelled, and the meeting and training were conducted virtually. Nevertheless, the implementation of the activities was successfully carried out even if the timeline had been extended.”
Indonesia	“Most of the effects were on data collection as most institutions, hospitals, and pharmacies were preoccupied with COVID-19–related works. Further, meetings with governmental staff were delayed as they had to give more attention to COVID-19 works.”
Lao PDR	“COVID-19 caused limited accessibility to the facility for face-to-face meetings, consultations, and data collection as we have a massive limitation of electronic data, causing a delay in data collection. We had to reduce travelling and collect data for the facilities located only in the capital city.”
Nepal	“COVID-19 restrictions forced us to have virtual communication with hospitals and labs, which was ineffective as it was challenging to establish professionalism over the phone and convince them to sign for data transfer agreement resulting in a lower participation rate. Researchers felt difficulty working in the hospital labs without proper distance maintenance and had to work in small and crowded spaces while taking precautions. During COVID-19, hospital staffs were very busy. Therefore, researchers had to reschedule the meetings frequently. Furthermore, due to the halt in activities caused by COVID-19, researchers at hospitals had to extend their working hours/days and put extra effort into completing the survey on time.”
Pakistan	“I joined during COVID-19, and this is one of the most streamlined and organised projects I have ever worked on.”
Sri Lanka	“Due to the change in the political situation, the government withdrew its support of Fleming Fund activities which was agreed upon by the previous officials. All data collected were returned to the government with the condition of not utilising them for any CAPTURA work. Due to COVID-19, there was a delay in meetings, and the deadline had to be pushed depending on the mitigation strategies. Face-to-face meetings switched to virtual settings. However, we conducted a 2-day WHONET workshop in a hybrid way with a very high participant number. Staff shortage was never an issue as we easily hired the required staff—no problems organising in-person meetings pre-, during, post-COVID. During COVID-19, the roads were less busy, so it was easier to travel for CAPTURA's data collection (from the government hospital sector before the government withdrew support). Doctors were all available at the hospital, so collecting data was accessible. Contrary to anyone's expectation, the COVID lockdown instead facilitated work than hindered it.”
Timor-Leste	“COVID-19 challenged us to organise in-person meetings as public transportation was not operated. In addition, remote work was difficult due to the slow but expensive Internet.”
**Question 2. Do you have suggestions for future projects to navigate through unforeseen circumstances such as a pandemic?**	
Bangladesh	“To accomplish this kind of initiative under these conditions, Bangladesh must have a powerful presence, as evidenced by stakeholder and facility-level participation. The CAPTURA project was entirely technology-driven, which might be improved in the following areas: To engage more technical persons during data collection and primary cleaning stage (for other countries). To introduce own IT platforms for metadata, data cleaning and visualisation though we did it for Bangladesh but not for other countries. To use central data storage and visualisation platform instead of a file-based data warehouse. Ensuring facility-level postsupport after implementing WHONET software. It could be the government stakeholders or the WHONET team. Providing more support to improve country ownership, especially on the WHONET, for further technical support. To define a minimal facility-level intervention such as antibiogram preparation of the facilities with the help of the CAPTURA project. We could implement an intervention that might be more impactful.”
Bhutan	“During unforeseen conditions in future, without waiting or delaying, we should resort to virtual meetings/workshops/training. Also, the risk mitigation measures should be mentioned and proposed so that we will have alternative plans to implement the activities.”
Indonesia	“As Government prefers to have such ownership, it would be great if the CAPTURA team could meet, introduce, and present the work in person in Indonesia rather [than] virtually. Also, understanding Indonesian bureaucracy on the international grant implementation would be an asset as a permission letter must be obtained before the project.”
Lao PDR	“Supporting the country to improve capacity for digitalised data collection and storage will help prevent future limitations and challenges.”
Nepal	“From our experience, we suggest having an updated list of contact directories for senior management at each hospital before beginning the project. It would enable me to efficiently reach out to the relevant hospital officials and resolve administrative issues. We also suggest the importance of sharing project details and providing group orientation to all beneficiary hospitals before drafting any memorandum of understanding or data transfer agreement with them. This helps to build trust and ownership among the hospitals and ensures that everyone is on the same page and working towards the same goals. Overall, these measures can reduce uncertainty and improve the efficiency and effectiveness of research activities in hospitals. However, despite receiving ethical clearance from Nepal Health Research Council, the researchers conducting the study faced several challenges when collecting data from individual facilities. Many of these facilities demanded the researchers go through their own Institutional Review Board, which was time-consuming and often needed additional fees to be paid despite already having paid 3% of their research budget to the NHRC. This lack of clarity and inconsistency in the ethical clearance process seriously hindered the researchers’ ability to collect data, and many facilities ultimately declined to participate in the study.”
Pakistan	“There was particular challenges in partnership management and accessing the data, which were resolved in time. CAPTURA previously also engaged and onboarded all partners from the project's conception to overcome that. This time they should be onboarded from the grant-application stage to ensure and maximise the ownership of the project of all key stakeholders.”
Sri Lanka	“In future, the CAPTURA team will have to be tactful in dealing with the bureaucratic system. CAPTURA officials should brief the top personnel in the Ministry/sector first and get to the focal point through them subsequently. The international party (such as IVI) could do that, while the country coordinator must follow the protocol. Thus, getting a written draft as an agreement from the start will be good. Another unforeseen issue is the reluctance of medical professionals due to a lack of trust in international organisations (other than WHO). They fear data misuse; therefore, getting them involved with transparent guidelines/ToR before the start of work would be beneficial.”
Timor-Leste	“Future projects could be switched to online platforms. Also, political changes should ensure the partnership management and obtaining an approval letter is completed on time.”
**Question 3. Do you have any other comments?**	
Bangladesh	“Cleaning and analysing facility-level data and creating the site-specific reports took much time. It may be reduced as data gathering was completed in May 2021, and report preparation required an additional year and a half, prolonging the results and decision-making time. We hope to faster up our next engagement.”
Bhutan	“Since the CAPTURA has completed the first stage of the project, which was successful in Bhutan, and the country report is being finalised, we would like to know if there is any plan from the CAPTURA team to disseminate the report or any way forward on the findings from the country.”
Pakistan	“Congratulations on a commendable project in Pakistan.”
Sri Lanka	“I suggest an intern go through our activity reports written by all CAPUTRA Country Coordinators. It would be good to categorise works as pre-, during, and post-COVID periods and randomly go through them to record information.”
Timor-Leste	“I suggest continuing CAPTURA projects routinely as the outcomes benefit the country. CAPTURA can have a branch office in the country or representative or have a permanent country consultant.”

Abbreviations: CAPTURA, Capturing Data on Antimicrobial Resistance Patterns and Trends in Use in Regions of Asia; COVID-19, coronavirus disease 2019; IT, information technology; IVI, International Vaccine Institute; Lao PDR, Lao People’s Democratic Republic; NHRC, Nepal Health Research Council; ToR, Terms of Reference; WHO, World Health Organization.

## CONCLUSIONS

This article presents the impact of the COVID-19 pandemic on the activities of the CAPTURA project across different countries. The approach to AMR/AMU/AMC surveillance was similar in most countries during the initial phase; however, during the peak of the pandemic, country-specific tailored approaches were implemented factoring in local situations and public health measures in place, which resulted in successful project completion. Despite some delays caused by the COVID-19 pandemic, the project was able to adapt and sustain its activities through the intensified use of virtual platforms and tailored approaches to AMR/AMU/AMC surveillance in different countries. The fact that project staff were already established locally is judged to have played an important role in the ability to successfully adapt and sustain our activities, albeit in an altered fashion and with some delays. Through the intensified use of virtual platforms, we were able to communicate and continue engagement with stakeholders, training of staff, and data collection. As such, this article highlights the importance of an adaptive approach to tailored engagement with stakeholders while closely monitoring the local situation to continue successful project implementation during unforeseen circumstances such as an ongoing pandemic with substantial travel and movement restrictions.

## Notes

***Authors Contributions.*** S. G. and K. P. contributed equally to draft first version, analyse data and prepare final version of manuscript. H. B., D. F., M. G., M. H., N. M., B. P., M. J. S., and P. Y contributed equally to to coordinate country activities, answer surveys for the paper and country-context of the paper. F. M. and M. H. contributed equally to to conceptualizing, planning, and overseeing the entire study.

***Acknowledgments.*** The authors are grateful to Jihyun Han and Hyein Yoon of the International Vaccine Institute (IVI) for programmatic support. Also, we highly appreciate Dr Ayesha Rasheed, who supported the CAPTURA in-country team in Pakistan, as well as Dr Amit Bhandari and Dr Reena Mukhiya in Nepal.

***Disclaimer.*** The views expressed here are those of the authors and not necessarily those of the UK Department of Health and Social Care or its management agent, Mott MacDonald; and, International Vaccine Institute.

***Supplement sponsorship.*** This article appears as part of the supplement “Capturing Data on Antimicrobial Resistance Patterns and Trends in Use in Regions of Asia (CAPTURA),” funded by the Department of Health and Social Care's Fleming Fund using UK aid.

***Financial support.*** This work was supported by the Fleming Fund regional grants (grant numbers FF10-135 and FF11-139). The Fleming Fund is a UK aid investment to support surveillance of antimicrobial resistance in low- and middle-income countries in Africa and Asia. The regional grants program is managed by Mott MacDonald Ltd, the Fleming Fund Grants Management Agent.

## References

[1] World Health Organization . Antimicrobial resistance. 2021. Available at: https://www.who.int/news-room/fact-sheets/detail/antimicrobial-resistance. Accessed 20 August 2023.

[2] Seale AC , Gordon NC, Islam J, Peacock SJ, Scott JAG. AMR surveillance in low and middle-income settings—a roadmap for participation in the Global Antimicrobial Surveillance System (GLASS). Wellcome Open Res 2017; 2:92. 29062918 10.12688/wellcomeopenres.12527.1 PMC5645727

[3] Tomczyk S , Taylor A, Brown A, et al Impact of the COVID-19 pandemic on antimicrobial resistance (AMR) surveillance, prevention and control: a global survey. J Antimicrob Chemother 2021; 76:3045–58. 34473285 10.1093/jac/dkab300 PMC8499888

[4] CAPTURA Project . Capturing Data on Antimicrobial Resistance Patterns and Trends in Use in Regions of Asia. 2022. Available at: https://captura.ivi.int/about. Accessed 24 June 2023.

